# Simulation Study on 6.5 kV SiC Trench Gate p-Channel Superjunction Insulated Gate Bipolar Transistor

**DOI:** 10.3390/mi16070758

**Published:** 2025-06-27

**Authors:** Kuan-Min Kang, Jia-Wei Hu, Chih-Fang Huang

**Affiliations:** Institute of Electronics Engineering, National Tsing Hua University, Hsinchu 30010, Taiwan; s107063543@m107.nthu.edu.tw (J.-W.H.); cfhuang@ee.nthu.edu.tw (C.-F.H.)

**Keywords:** SiC, trench gate, IGBT, superjunction, forward voltage, turn-off energy loss

## Abstract

This paper investigates 6.5 kV SiC trench gate p-channel IGBTs using Sentaurus TCAD simulations. The proposed superjunction structure is compared to conventional designs to highlight its advantages. The p-IGBT, fabricated on an n-type substrate, offers notable commercial advantages over n-IGBTs on p-type substrates. The n-shield can effectively protect the trench gate oxide in the corners of SiC. The n-shield and n-pillar can be either floating or grounded, with the floating shield condition significantly enhancing injection and improving forward conduction performance. The superjunction floating shield p-IGBT (SJFS-p-IGBT) improves forward conduction voltage (V_F_) by 47% and 15% compared to conventional planar gate p-IGBT (CP-p-IGBT) and grounded shield p-IGBT (CGS-p-IGBT), respectively. For switching characteristics, the superjunction grounded shield p-IGBT (SJGS-p-IGBT) improves turn-off time (t_off_) by 15% compared to the conventional floating shield p-IGBT (CFS-p-IGBT). The trade-off between V_F_ and turn-off energy (E_off_) is analyzed, showing that the SJFS-p-IGBT offers a better trade-off. A negative temperature coefficient is observed at high buffer layer doping concentration and elevated temperatures, leading to an increase in V_F_. This provides design guidance for devices operating in parallel at high temperatures. These results demonstrate the SJ’s potential to enhance efficiency and performance for ultra-high voltage applications.

## 1. Introduction

High-voltage and high-current IGBTs are primarily designed for medium to ultra-high-power applications such as industrial applications, switched-mode power supplies, and traction systems [1,2]. Silicon IGBTs have been utilized and approached their inherent limits [3,4,5]. Furthermore, their development in these applications is significantly constrained by material limitations in operating frequency, voltage rating, and temperature. As a wide bandgap material, power devices in SiC have gained considerable interest due to their superior material properties [6,7]. From several hundred volts to approximately 3.3 kV, SiC MOSFET has been widely used and commercialized [8,9]. However, for ultra-high-voltage applications exceeding 6.5 kV, due to a significant reduction in conduction loss by conductivity modulation, bipolar devices, such as IGBTs and thyristors, are favored over unipolar devices in 4H-SiC [10,11]. On the other hand, IGBTs show some advantages over thyristors. Firstly, an IGBT is controlled by the voltage at the gate, similar to a MOSFET, offering a high-input impedance with low driving power. Secondly, IGBT typically supports switching frequencies up to tens of kHz or even higher, while thyristors are typically used below several hundred Hz. Thus, except for some ultra-high-power applications at low frequencies, most medium to high applications are dominated by IGBTs. Several studies have shown that superjunction (SJ) IGBT on Si has better turn-off energy (E_off_) and forward conduction voltage (V_F_) performance than conventional IGBT [12,13,14], resulting from the electric field distribution two-dimensional depletion expansion in the pillars. At the same time, charge balance in n- and p-pillars enables a thinner drift region thickness, which also helps improve storage charge for better E_off_ and V_F_ [15,16]. The investigation on SiC 6.5 kV trench gate p-IGBT is proposed for the first time in this work. P-IGBTs feature two distinct advantages over n-IGBTs in SiC. Firstly, they are economically more feasible primarily due to the lack of good conducting p-type SiC substrates. Secondly, p-IGBTs exhibit higher transconductance than n-IGBTs because the parasitic npn transistor in p-IGBTs has a higher common base current gain than the pnp transistor in n-IGBTs [17,18]. In the meantime, the trench gate structure is used to reduce the cell pitch size and increase channel density [19], counteracting the deficiency in poor p-channel mobility in SiC. In this paper, SiC 6.5 kV trench gate superjunction and conventional p-IGBTs are investigated using TCAD two-dimensional simulations. The E_off_ and V_F_ trade-offs with respect to critical parameters such as the buffer layer, the carrier lifetime, and the temperature are examined. Physics models include Shockley–Read–Hall (SRH), Auger, Okuto–Crowell avalanche model, doping-dependent mobility, high-field saturation mobility, and Incomplete Ionization [20]. Based on the results, 6.5 kV-class SiC trench gate superjunction p-IGBTs could be a strong candidate for power switches in ultra-high-power applications [21,22,23].

## 2. Device Structure

**Figure 1:**
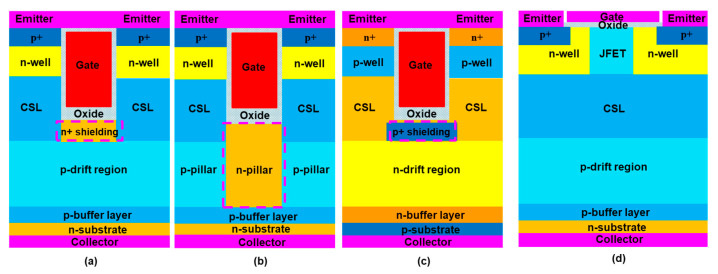
Schematic cross-section of (**a**) a conventional trench gate p-IGBT, (**b**) a superjunction trench gate p-IGBT, (**c**) a conventional trench gate n-IGBT, and (**d**) a conventional planar gate p-IGBT. The dashed n,p-shield region and n-pillar can be grounded or floating.

**Figure 2:**
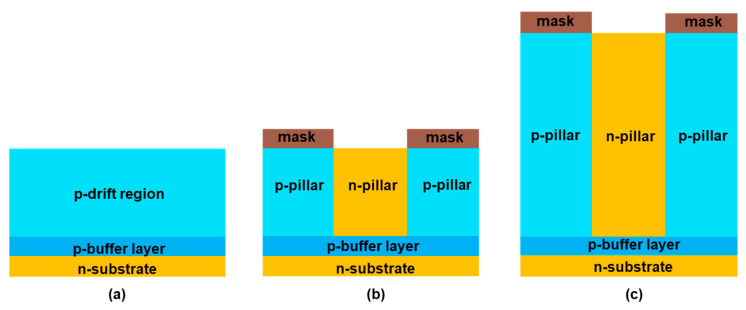

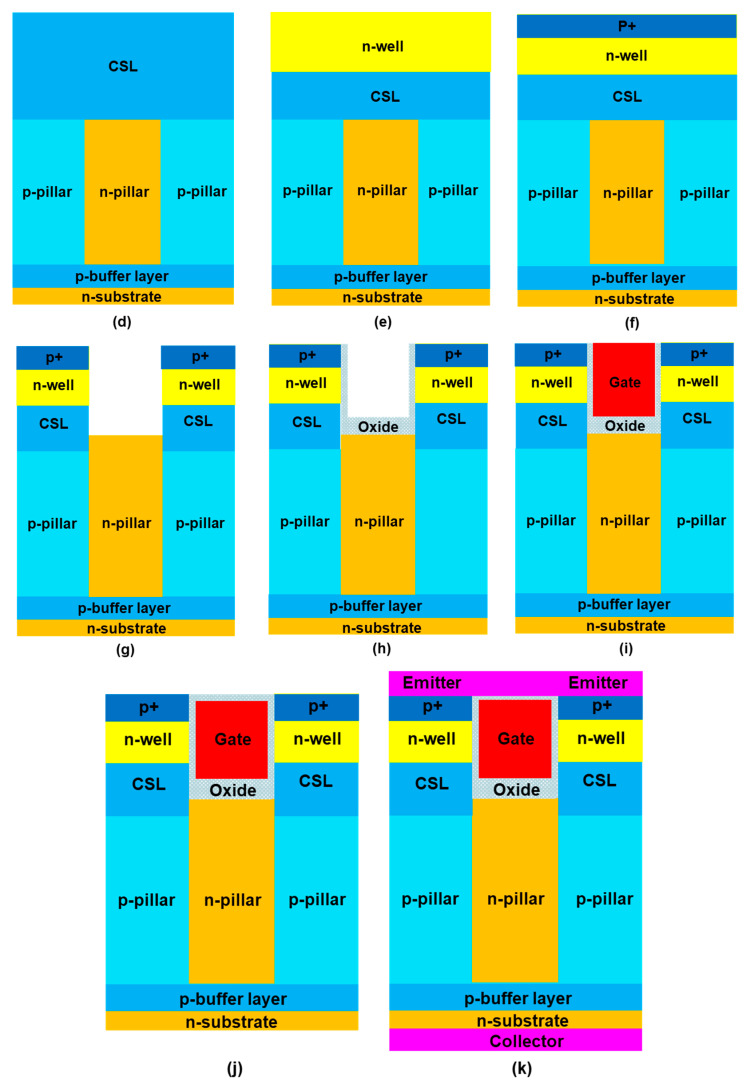
Schematic fabrication process of SJ trench gate p-IGBT. (**a**) The p-drift region and p-buffer layer are formed on the n-substrate using epitaxial deposition technique; (**b**) phosphorus ion implantation to form n-pillars; (**c**) repeat ME technique for 30 times to form the SJ structure; (**d**) aluminum ion implantation to form CSL; (**e**) phosphorus ion implantation to form n-well; (**f**) aluminum ion implantation to form p+; (**g**) trench etch; (**h**) gate oxide deposition; (**i**) polysilicon deposition to form the gate; (**j**) inter-layer dielectric formation; (**k**) contact formation and metal deposition.

Figure 1 shows the cross-sectional view of a conventional trench gate p-IGBT, a trench gate superjunction p-IGBT, a conventional trench gate n-IGBT, and a planar gate p-IGBT in 4H-SiC. In the trench gate structures, the n-well is always connected through an n+ region to the emitter electrode in the third dimension to reduce cell pitch. In simulation, this is achieved by placing a virtual electrode on the side of the n-well at an appropriate location, similar to our previous study [24]. The n-shield region and n-pillar can be either grounded or floating in the third dimension, which can be realized through appropriate layout designs. The doping concentration and the thickness of the drift region are 1 × 10^15^ cm^−3^ and 45 μm, respectively, for a 6.5 kV-rated blocking voltage for conventional IGBTs [25]. For the SJ-IGBTs, the pillar doping concentration is about 1 × 10^15^ cm^−3^ for both n- and p-pillars. The pillar widths are 1 and 1.2 μm for n-pillar and p-pillar, respectively. With aggressive scaling, the cell pitch is assumed to be 2.2 μm, including a 1 μm-wide gate trench, and the trench depth is 1.5 μm. All the device parameters are chosen based on the previously reported results and the current capabilities of foundries. If the cell pitch is aggressively scaled to less than 2.2 μm, then V_F_ starts to increase due to significant junction field-effect transistor (JFET) effect. The gate oxide layer is uniform and 42 nm thick. To realize high-performance SiC superjunction (SJ) structures presents considerable challenges, primarily due to the need for advanced fabrication techniques and precise doping control to achieve superior device performance [26,27]. In this study, a multi-epitaxial (ME) growth technique is employed [28], which involves repeated cycles of epitaxial layer deposition and ion implantation to form n-pillars. To serve as an electric field stopping layer and to prevent punch-through of the drift region in the blocking state, a buffer layer with 2.5 μm thickness is applied. The channel length is 0.5 μm, and the inversion hole mobility is assumed to be around 12 cm^2^/V-s based on previously reported numbers [29,30]. The depth of the shielding in the conventional grounded shied p-IGBT (CGS-p-IGBT) and the conventional floating shied p-IGBT (CFS-p-IGBT) is 1.1 μm. To enhance forward conduction characteristics, a current spreading layer (CSL) is added on top of the drift layer [31,32]. Regarding the forward conduction operation, a JFET effect will occur between n-well and n+ shielding or adjacent n+ shielding regions, leading to an increase in specific on-resistance (R_on,sp_). The CSL is a p-type region with a higher concentration than the drift region, providing more holes during forward conduction, thereby benefiting the conduction characteristics. The doping concentration of the CSL is 1 × 10^16^ cm^−3^. The doping concentration of the n-substrate region is 1 × 10^19^ cm^−3^. The carrier lifetime is assumed to be 1 μs unless indicated otherwise. The conventional trench gate grounded shied n-IGBT (CGS-n-IGBT) is designed with the same dimensions and doping concentrations as the CGS-p-IGBT, except for the opposite n-type and p-type dopants. The p-type substrate is assumed to be very thin to remove its resistance contribution. All the SJ devices have the same dimensions and doping concentrations above the CSL and the trench oxide as the conventional device, except for the drift region to support a fair comparison. The fabrication process schematic of the ME growth SJ trench gate p-IGBT is shown in Figure 2. (a) The p-drift region and p-buffer layer are formed on the n-substrate using epitaxial deposition technique; (b) phosphorus ion implantation to form n-pillars; (c) repeat ME technique for 30 times to form the SJ structure; (d) aluminum ion implantation to form CSL; (e) phosphorus ion implantation to form n-well; (f) aluminum ion implantation to form p+; (g) trench etch; (h) gate oxide deposition; (i) polysilicon deposition to form the gate; (j) inter-layer dielectric formation; (k) contact formation and metal deposition.

## 3. Static Characteristics

**Figure 3:**
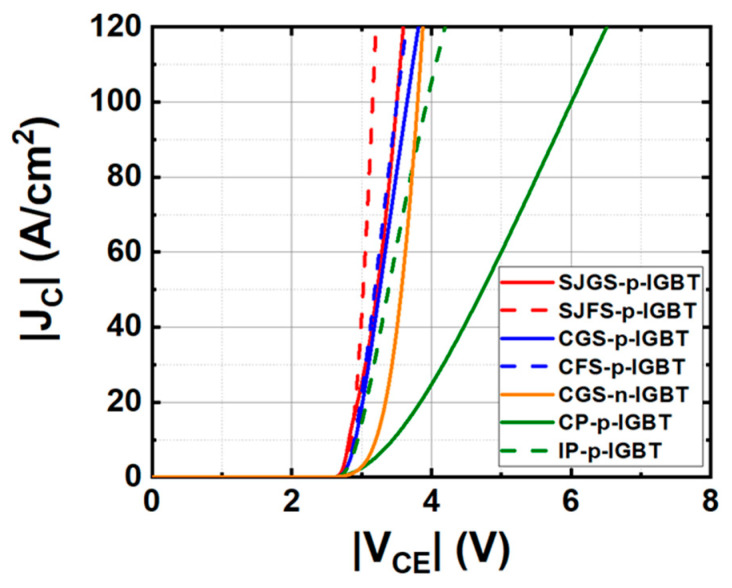
Simulated J_C_–V_CE_ characteristics of the studied IGBTs.

Figure 3 illustrates the forward J_C_–V_CE_ characteristics at a V_GE_ of −16 V for the trench gate p-IGBTs under study. For comparison, a CGS-n-IGBT, the n-channel mobility is set to be around 30 cm^2^/V-s based on previously reported numbers in [33], and a conventional planar gate p-IGBT is also simulated based on the structure experimentally demonstrated in [31], denoted as conventional planar gate p-IGBT (CP-p-IGBT), where the cell pitch is 15 μm, including a 3 μm JFET. The drift region, as well as the CSL doping and thickness, are adjusted to be the same as those of the trench gate IGBT structure for the same voltage rating. An improved planar gate p-IGBT (IP-p-IGBT) is also included in this study with a reduced cell pitch of 4.4 μm, including a JFET width of 2.2 μm, channel length of 0.5 μm, and 1.2 μm p+ region width. In this case, the n+ region is connected in the third dimension, similar to the trench gate structure, to aggressively scale down the cell pitch of the planar gate IGBT. Further reducing the JFET width and the cell pitch will degrade performance due to pinch-off by the JFET effect. It should be noted that the cell pitch of a planar gate structure cannot be as small as a trench gate structure due to the lateral channel length and JFET width. The detailed device structure parameters are provided in Table 1.

From Figure 3, the simulated V_F_ of CP-p-IGBT and IP-p-IGBT reaches as high as −6 V and −4.2 V, respectively, at J_C_ = −100 A/cm^2^, while the V_F_ values of the trench gate IGBTs are all below −4 V. Due to the poor p-channel hole mobility in SiC, a large cell pitch, as in the case of a planar gate p-IGBT, limits the hole current density, thereby reducing the back-injected electron density into the drift region, which is unfavorable for conductivity modulation. On top of that, the poor p-channel resistance contributes a significant voltage drop to the V_F_. Since the cell pitch of the trench gate structure can be much smaller than that of the planar gate structure, the aforementioned drawbacks from poor p-channel mobility can be minimized. If we look further into the V_F_ values of CGS-p-IGBT, CFS-p-IGBT, SJGS-p-IGBT, SJFS-p-IGBT, and CGS-n-IGBT, they are −3.73 V, −3.57 V, −3.55 V, −3.20 V, and 3.82 V, respectively, comparable to reported simulation results for SiC n-channel IGBTs [33,34]. The first conclusion we can make at this point is that with a trench gate structure and a small cell pitch, the DC characteristics of SiC p-IGBTs are comparable to SiC n-IGBTs.

**Figure 4:**
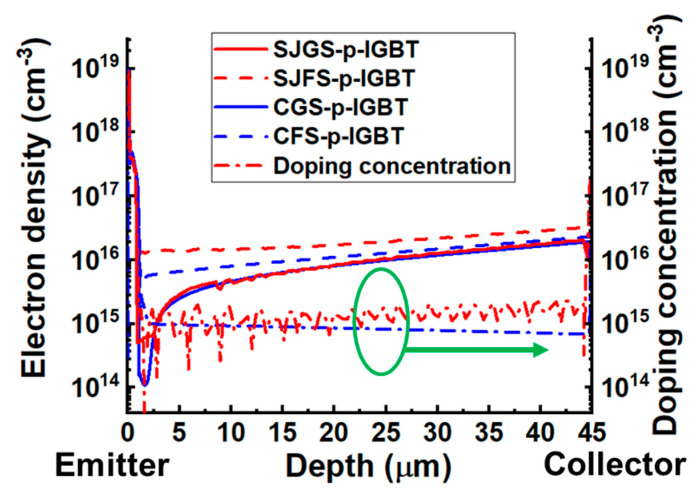
Distribution of electron density and doping concentration along the drift region from emitter to collector in the drift region of the investigated trench gate p-IGBTs.

In general, the floating shield has superior forward characteristics. The increase in V_F_ with the grounded n-shield is caused by the reduction in minority carrier concentration because the grounded shield enables the extraction of minority carriers, which are electrons, with an external electrode. Conversely, the floating shield has better conductivity modulation since electrons are stored at the upper side of the drift region. Figure 4 shows the distribution of electron density and doping concentration along the drift region from emitter to collector in the investigated trench gate p-IGBTs when applying V_GE_ = −16 V and V_CE_ = −4 V. Thus, the V_F_ of the floating shield condition is lower than that of the grounded shield condition in both cases, consistent with [33]. The V_F_ of SJFS-p-IGBT is reduced by 15% compared to that of CGS-p-IGBT and further reduced by 28% compared to that of IP-p-IGBT.

**Figure 5:**
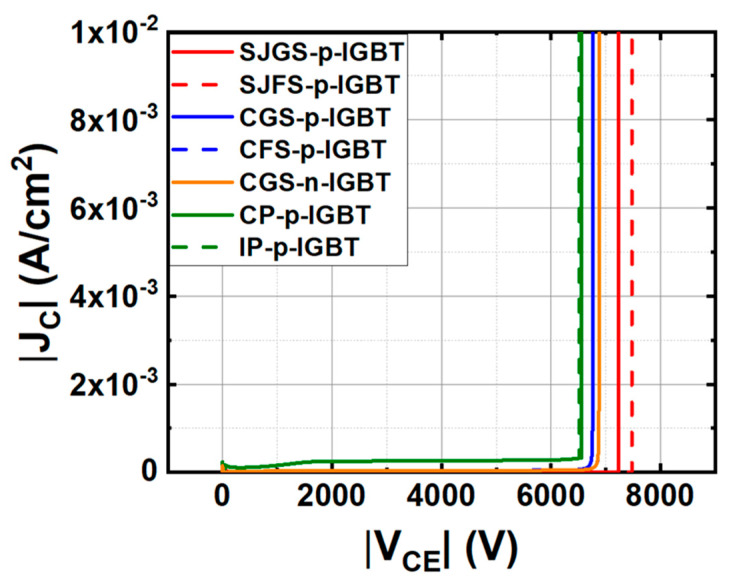
Blocking characteristics (V_GE_ = 0 V) of the studied IGBTs.

**Figure 6:**
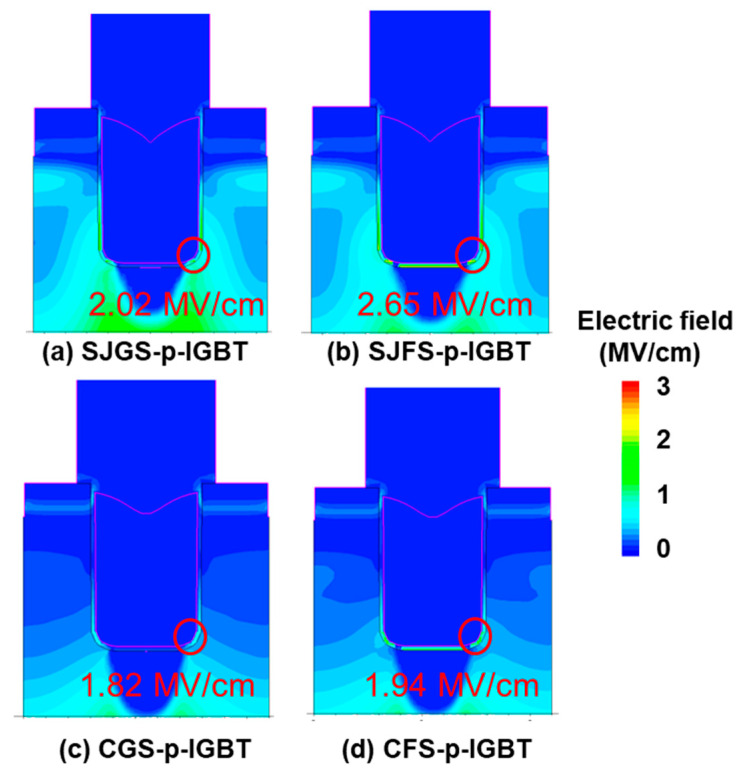
Electric field distribution in the off-state for the studied trench gate p-IGBTs at V_CE_ = −6.5 kV and V_GE_ = 0 V.

Figure 5 shows the simulated breakdown characteristics (V_GE_ = 0 V) of all the structures. The BV values of all devices are above the targeted 6500 V, and the leakage currents are all below 1 × 10^−3^ A/cm^2^. Figure 6 illustrates the electric field distribution of all trench gate devices at V_CE_ = −6500 V. The electric field at the bottom corners of the gate trench does not exceed 3 MV/cm in all cases, indicating that the n-shield region and n-pillar effectively mitigate the electric field in the oxide and preserve gate oxide integrity. The discussion in the following sections will focus on the proposed trench gate p-IGBT structures to further explore their switching performance to justify their potential in real applications.

## 4. Dynamic Characteristics

**Figure 7:**
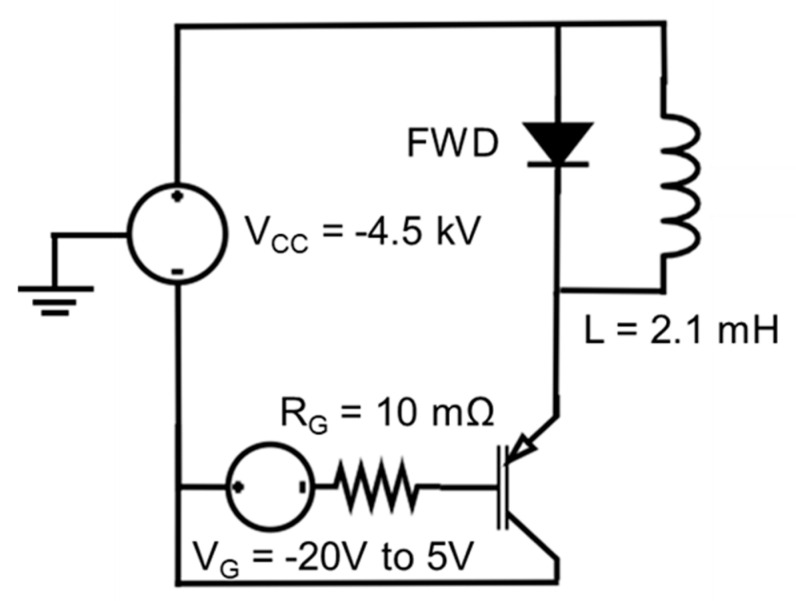
A clamped inductive load circuit for the switching characteristics of the studied IGBTs with an active area of 68 mm^2^.

**Figure 8:**
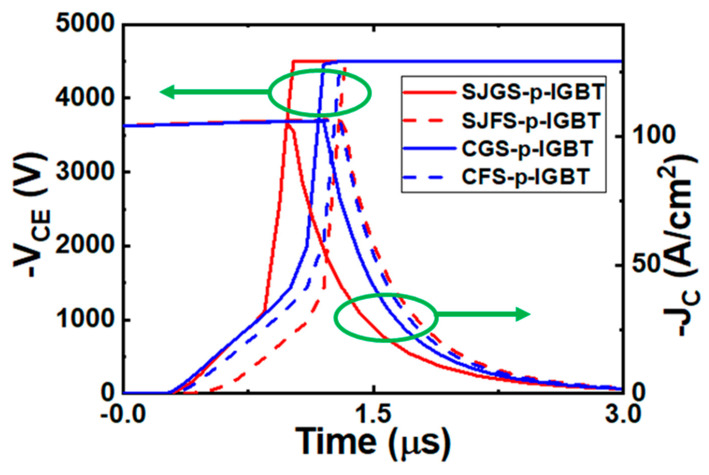
Turn-off voltage and current waveforms of the studied IGBTs.

The switching characteristics of the IGBTs are investigated using a double pulse test circuit shown in Figure 7. The supply voltage is V_CC_ = −4.5 kV, and the gate voltage V_G_ changes from −20 V to 5 V to turn the device on and off, respectively. The active area of the IGBT is scaled to 68 mm^2^. The load inductance is 2.1 mH, and an external gate resistor R_G_ = 10 mΩ is used. The turn-off waveforms for all the structures are shown in Figure 8. The SJGS-p-IGBT exhibits a slightly shorter turn-off time (t_off_). Since the electron density near the shield region in conduction is lower than that of both the SJFS-p-IGBT and CFS-p-IGBT, the V_CE_ potential builds up quickly at the beginning of the turn-off transient, which leads to shorter t_off_.

**Figure 9:**
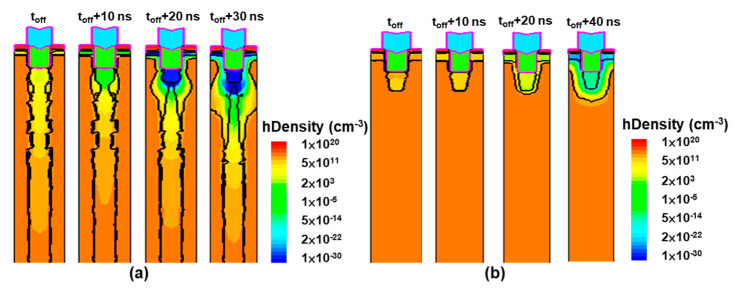
Hole density distribution and depletion region (denoted as the black line) during turn-off periods of (**a**) the SJGS-p-SJBT and (**b**) the CGS-p-IGBT.

Moreover, the t_off_ values of SJGS-p-IGBT, SJFS-p-IGBT, CGS-p-IGBT, and CFS-p-IGBT are calculated to be 1.7, 2, 1.9, and 2 μs, respectively. Generally, the superjunction structure has a slightly shorter t_off_ due to the drift region being fully depleted faster because of the two-dimensional expansion of the depletion region. Figure 9 illustrates the depletion region and the hole density distribution during the turn-off periods of SJGS-p-IGBT and CGS-p-IGBT. At 30 ns after turn-off, the depletion region in the SJGS-p-IGBT expands well into the drift region with the lateral depletion from pillars, while at 40 ns in the CGS-p-IGBT, the depletion region remains around the n-shield. Thus, a better t_off_ and E_off_ is expected from this effect.

It is noted that before 1 kV, the V_CE_ increases slowly. Since the electron density is higher at the collector side of the drift region, more excess carriers need to be removed to achieve the same voltage buildup. Thus, the V_CE_ increases slowly during this stage of the turn-off transient. This process is faster for the superjuction structure for the reason explained previously. After 1 kV, as the depletion region starts to extend into the buffer region where the doping concentration is higher, the electric field accumulates more rapidly, leading to a faster increase rate for V_CE_. Eventually, when V_CE_ reaches the punch-through voltage, the collector current then decreases until it reaches zero, completing the turn-off process [33,35]. Different from the turn-off process, the differences in the turn-on waveform and turn-on energy are not significant among the IGBTs under study and, therefore, are not presented in this paper.

## 5. Key Performance Parameters

### 5.1. Carrier Lifetime

**Figure 10:**
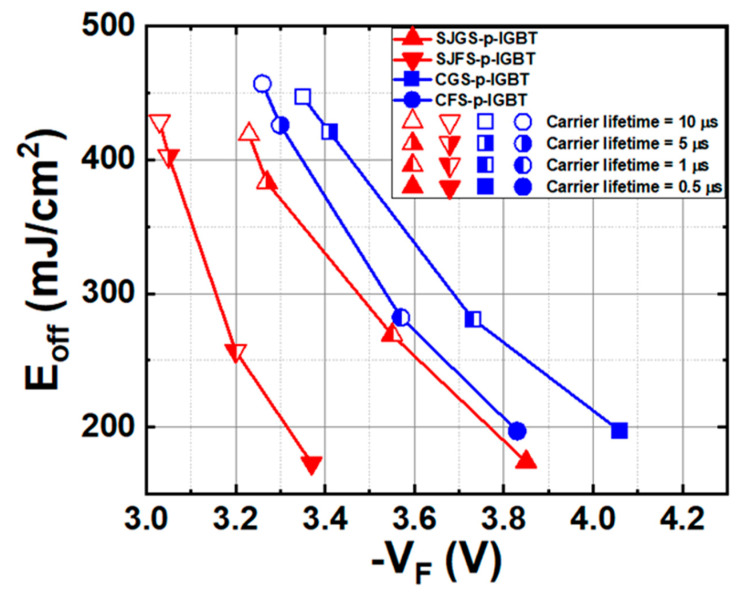
Trade-off curves of V_F_ and E_off_ of the studied IGBTs with different carrier lifetimes.

To reduce switching loss, carrier lifetime control in the drift region is important. Due to the conductivity modulation of IGBTs, the drift region resistance primarily depends on the carrier lifetime in the drift region [36,37]. High-voltage bipolar devices require a long carrier lifetime to effectively modulate the conductivity of the thick drift region layer. In contrast, a short carrier lifetime hinders conductivity modulation, making the forward voltage high. Conversely, a long carrier lifetime will lead to a long reverse recovery time, which limits the switching frequency and increases switching loss [38]. Therefore, the carrier lifetime must be carefully controlled to achieve optimal performance. Figure 10 shows the trade-off between turn-off energy loss and forward voltage drop for all the structures. It is observed that the SJFS-p-IGBT shows the best V_F_-E_off_ trade-off while the CGS-p-IGBT shows the worst trade-off among the IGBTs under study.

### 5.2. Buffer Layer Doping Concentration

**Figure 11:**
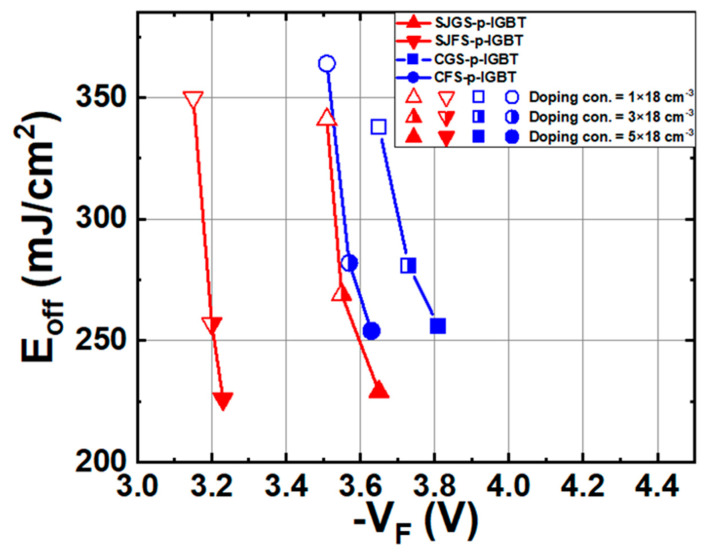
Trade-off curves of V_F_ and E_off_ of the studied IGBTs with various buffer layer doping concentrations.

The buffer layer concentration has a great impact on both the stored charge and the switching speed, and the mechanism is clearly explained in [39,40]. Figure 11 shows the trade-off between E_off_ loss and V_F_ as the buffer layer concentration increases from 1 × 18 cm^−3^ to 5 × 18 cm^−3^. The E_off_ decreases dramatically because the carrier lifetime of the buffer layer decreases as the concentration increases, based on a concentration-dependent lifetime. The ambipolar diffusion length in the buffer layer becomes shorter than the buffer layer thickness, reducing the injection efficiency of electrons into the drift layer [39]. As a result, the V_F_ increases. The SJFS-p-IGBT shows the best V_F_-E_off_ trade-off. Conversely, the CGS-p-IGBT exhibits a much worse V_F_-E_off_ trade-off in comparison with the other IGBTs.

### 5.3. Temperature Effect

**Figure 12:**
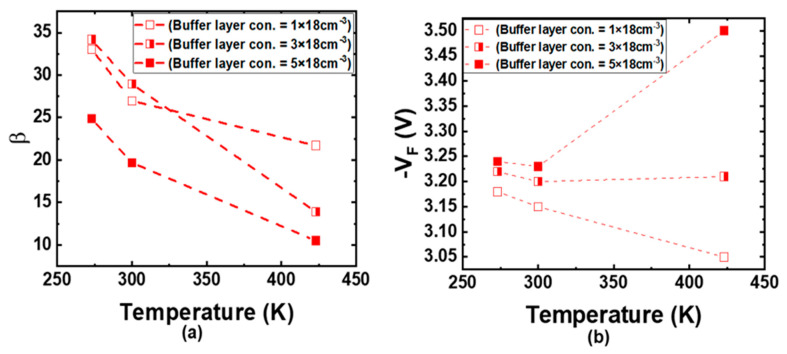
The temperature effect of the buffer layer doping concentration in a SJFS-p-IGBT on (**a**) common emitter current gain (β) for the parasitic npn BJT and (**b**) forward voltage at J_C_ = −100 A/cm^2^.

The equivalent circuit model of a p-IGBT consists of a wide-base npn BJT and a p-channel MOSFET. Several studies [41,42,43,44] have indicated that a 4H-SiC npn BJT can show either positive or negative temperature coefficients for its common emitter current gain (β). The positive temperature coefficient is attributed to the increase in the carrier lifetime as the temperature rises. On the other hand, the negative temperature coefficient results from the incomplete ionization of acceptors in the base region [45]. With the temperature increasing, the hole concentration will increase, leading to a lower injection efficiency, and it is worse for higher p-type concentrations. Thus, the 4H-SiC npn BJT can have a lower current gain at a higher temperature. The p-buffer layer in the p-IGBT structure plays the same role and affects the emitter injection efficiency in the parasitic npn BJT. Figure 12 illustrates the β and V_F_ of the SJFS-p-IGBT at a collector current of 100 A/cm^2^ at different temperatures for different buffer layer doping concentrations. A smaller current gain will be obtained at a higher temperature for all buffer layer doping concentrations, and this is more pronounced for >3 × 18 cm^−3^. It is shown that the V_F_ decreases monotonically as the temperature increases in the case of 1 × 18 cm^−3^. However, as the buffer layer doping concentration is greater than 3 × 18 cm^−3^, the V_F_ starts to show a positive temperature coefficient at a higher temperature, due to the reduced injection of minority carrier electrons from the n-substrate in this case. Hence, the buffer layer doping concentration must be considered when a positive temperature coefficient in V_F_ is preferred for paralleling 4H-SiC p-IGBT chips.

## 6. Conclusions

In summary, the investigation on a novel SiC 6.5 kV trench gate p-IGBT with a superjunction structure is proposed in this work. The p-IGBT demonstrates greater commercial potential due to the adoption of a low-resistivity n-type substrate, which facilitates cost-effective manufacturing. The V_F_ and E_off_ trade-off of the device is examined by varying the buffer layer concentration, carrier lifetime, and temperature. From the simulated static characteristics, the SJFS-IGBT can improve the V_F_ by 15%, relative to the CGS-p-IGBT. For the switching characteristics, the SJFS-p-IGBT demonstrates 15% improvement in t_off_, compared to the CFS-p-IGBT. Regarding the temperature effect, depending on the buffer layer doping concentration, all the structures exhibit noticeably negative temperature coefficients on the current gain, particularly when the buffer layer doping concentration is higher than 3 × 18 cm^−3^. The reduced injection efficiency will turn the temperature coefficient from negative to positive, especially for high buffer layer doping concentrations and high temperatures. The results presented in this paper pave the way for the 4H-SiC trench gate p-IGBT with superjunction for ultra-high voltage applications.

## Figures and Tables

**Table 1:** Device parameters of the IGBTs.

Parameters	Conventional Planar Gate p-IGBT	Improved Planar Gate p-IGBT	Conventional Trench Gate n-IGBT	Conventional Trench Gate p-IGBT	Superjunction Trench Gate p-IGBT
Buffer layer depth (μm)	2.5	2.5	2.5	2.5	2.5
Buffer layer doping (cm^−3^)	1 × 10^18^	1 × 10^18^	1 × 10^18^	1 × 10^18^	1 × 10^18^
Drift region depth (μm)	45	45	45	45	45
Drift region doping (cm^−3^)	1 × 10^15^	1 × 10^15^	1 × 10^15^	1 × 10^15^	1 × 10^15^
CSL doping (cm^−3^)	1 × 10^16^	1 × 10^16^	1 × 10^16^	1 × 10^16^	1 × 10^16^
Channel length (μm)	1.5	0.5	0.5	0.5	0.5
JFET width (μm)	3	2.2	--	--	--
Cell pith (μm)	15	4.4	2.2	2.2	2.2

## Data Availability

The original contributions presented in the study are included in the article, further inquiries can be directed to the corresponding author.
